# Insulin‐like growth factor 2: a poor prognostic biomarker linked to racial disparity in women with uterine carcinosarcoma

**DOI:** 10.1002/cam4.1335

**Published:** 2018-02-18

**Authors:** Anne R. Van Arsdale, Rebecca C. Arend, Maria J. Cossio, Britt K. Erickson, Yanhua Wang, David W. Doo, Charles A. Leath, Gary L. Goldberg, Gloria S. Huang

**Affiliations:** ^1^ Albert Einstein College of Medicine and Montefiore Medical Center Bronx New York; ^2^ University of Alabama at Birmingham Birmingham Alabama; ^3^ University of Minnesota Minneapolis Minnesota; ^4^ Northwell Health and Hofstra University Hempstead New York; ^5^ Yale University School of Medicine New Haven Connecticut

## Abstract

The objective of this study was to investigate the relationship of insulin‐like growth factor 2 (IGF2) expression and survival in women with uterine carcinosarcoma (UCS). Insulin‐like growth factor 2 protein expression was determined by immunohistochemical staining of tumor tissues from 103 patients with UCS. The H‐score (product of staining intensity and percentage positive cells) was quantified for the epithelial cytoplasmic (EC), epithelial nuclear (EN), and malignant stromal compartments. Multivariable Cox proportional hazard regression models were used to examine the relationship of IGF2 levels with progression‐free survival (PFS) and overall survival (OS). Adjusting for stage, race, and adjuvant therapy, PFS and OS were reduced in patients with high IGF2 (H‐score ≥ median) in the EC and EN compartments. Black race was independently associated with reduced PFS and OS in patients with early‐stage disease, and IGF2 levels in the EC were higher in black than in white patients (*P *=* *0.02, Wilcoxon test). In a race‐stratified multivariable analysis, high IGF2 in the epithelial compartments more than doubled the risk of death in black women; HR = 2.43 (95% CI: 1.18–5.01, *P *=* *0.02) for high IGF2 in the EC; and HR = 2.34 (95% CI: 1.25–4.39, *P *=* *0.008) for high IGF2 in the EN. In conclusion, high tumor IGF2 expression is an independent risk factor for reduced PFS and OS in UCS. Black women have elevated tumor IGF2 compared with white women, and decreased survival associated with high IGF2. These findings identify IGF2 as a candidate biomarker for survival linked to racial disparity in women with UCS.

## Introduction

Uterine carcinosarcoma (UCS) is a rare but highly aggressive uterine malignancy that accounts for a disproportionate number of endometrial cancer deaths 1. These biphasic tumors are composed of carcinomatous and sarcomatous elements originating from a common progenitor cell 2. Mutations in chromatin remodeling genes and histones are implicated in the tumorigenesis of carcinosarcoma, characterized by sarcomatous transformation of carcinoma cells via epithelial mesenchymal transition (EMT) 2, 3, 4, 5. Black women have more than a twofold higher incidence of UCS compared to white women, as well as higher mortality 6, 7, 8. Five‐year survival rates are approximately 30% for all women with UCS, and 50–60% for patients with disease confined to the uterus 9.

The insulin‐like growth factor 2 (*IGF2*) gene is located on chromosome 11 and is one of the few hundred imprinted genes in the genome. Imprinting is maintained by epigenetic mechanisms, mainly DNA methylation 10. *IGF2* promotors are developmentally regulated and selectively repressed in adult tissue types 11. *IGF2* upregulation has been observed in many childhood and adult malignancies. IGF2 overexpression occurs in the majority of sarcomas, often associated with loss of imprinting at the *IGF2* locus, and approximately 50% of uterine leiomyosarcomas have high IGF2 expression 12. Loss of *IGF2* imprinting has also been observed in UCS tumor tissue 13, and nonislet cell hypoglycemia due to tumor production of incompletely processed forms of pro‐IGF2 (“big” IGF2) was previously reported in patients with UCS 14, supporting a potential, unrecognized role of IGF2 in UCS.

The insulin‐like growth factor (IGF) family includes ligands (IGF1 and IGF2), tyrosine kinase receptors (IGF1R and IGF2R), and IGF binding proteins. The interaction between the ligands and the receptors is a complex, dynamic interplay 10, 15. IGF ligands as well as insulin bind to IGF receptors, insulin receptors, and hybrid receptors, leading to tyrosine autophosphorylation and activation. Receptor activation leads to phosphorylation of insulin receptor substrate and Src homology 2 domain‐containing transforming protein 1 (SHC). Phosphorylation of these receptor substrates results in activation of the phosphatidylinositide 3‐kinase/AKT (PI3K/AKT) and mitogen‐activated protein kinase (MAPK) signaling cascades 16. These signaling cascades inhibit apoptosis and promote cellular proliferation. Reactivation of fetal *IGF2* promoters and IGF2 overexpression is associated with worse prognosis in epithelial ovarian cancer and other cancers 17, 18, 19. Dysregulation of the IGF signaling pathway is linked to EMT, a major driver of metastasis and drug resistance and a defining feature of UCS 15.

The objective of our study was to evaluate tumor IGF2 expression in a cohort of patients with UCS and determine the relationship of IGF2 with clinicopathologic risk factors, and with disease progression and survival.

## Methods

This study was reviewed and approved by the Institutional Review Boards of the Albert Einstein College of Medicine and the University of Alabama at Birmingham. Formalin‐fixed, paraffin‐embedded (FFPE) tumor specimens were retrieved from 103 uterine CS patients undergoing primary surgical treatment.

Medical records were abstracted including date of diagnosis, stage, age, race/ethnicity, body mass index in kg/m^2^ (BMI), adjuvant therapy (chemotherapy, radiation therapy, or both), disease recurrence or progression, and date of death. Entry date for this analysis was defined as the date of histopathological diagnosis. The date of death was obtained by review of medical records and review of the Social Security Death Index.

### Immunohistochemistry

Immunohistochemistry was performed on FFPE tissue sections (one case per glass slide) using a rabbit polyclonal antibody directed at the IGF2 ligand (AB9574; Abcam Inc., Cambridge, MA). The optimized protocol has been previously validated and described 17. In brief, Target Retrieval Solution, Citrate pH 6 (Dako North America, Inc.) was used for antigen retrieval, TBS containing 5% goat serum and 2% bovine serum albumin was used for blocking, and the primary antibody was used at a 1:100 dilution with an incubation of 1 h at room temperature. Secondary antibody and detection were performed using the Dako Envision+ Polymer System (Dako North America), followed by counterstaining with hematoxylin. Staining of all tissue slides was performed concurrently with staining of positive and negative control sections.

**Figure 1:**
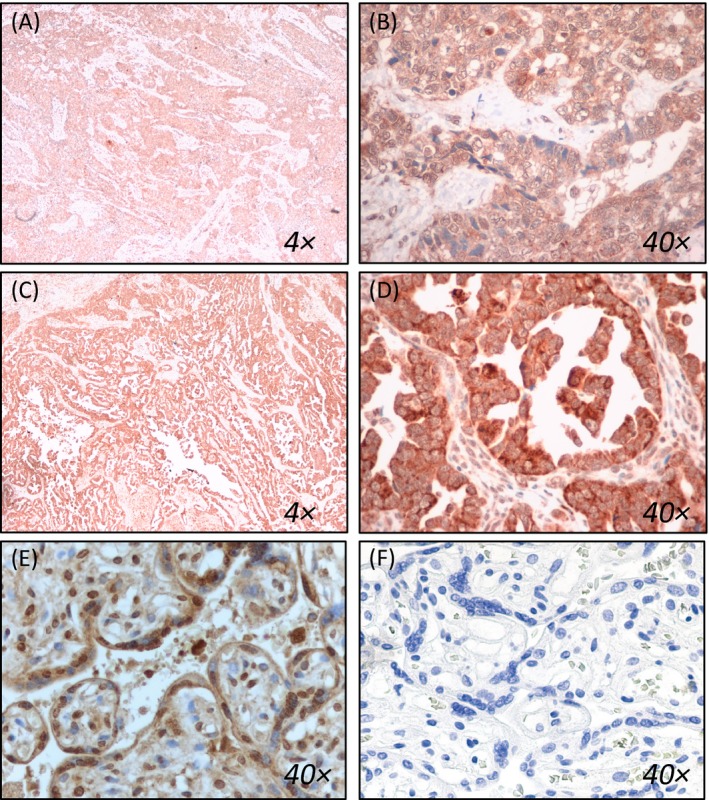
IGF2 immunohistochemical staining in uterine carcinosarcoma. Representative images of IGF2 immunohistochemistry are shown. In A (4x obj.) and B (40x obj.), the uterine carcinosarcoma tissue shows low IGF2 expression in all of the tumor compartments (EN, EC, SN, and SC). In contrast, the UCS tissue shown in C and D had high IGF2 expression in the EC and SC, but absent IGF2 expression in both nuclear compartments (EN and SN). Positive and negative control tissue sections are shown in E and F.

Representative‐stained carcinosarcoma tissue sections were photographed on a Zeiss Axioskop II, and images shown in Figure 1 depict the TIFF image files without modifications, other than cropping of the size. The study pathologist (Y.W.), who was blinded to all clinical data, evaluated the IGF2 staining intensity (0 negative, 1+ weak, 2+ moderate, 3+ strong) and the percentage of tumor cells with positive staining (1–100%) in each tumor compartment (epithelial cytoplasmic, epithelial nuclear, stromal cytoplasmic, and stromal nuclear). IGF2 expression was summarized using the H‐score, which is defined as the product of the staining intensity and the percentage of positive staining cells.

### Statistical analysis

Patient demographics and clinical characteristics were described as appropriate for continuous and categorical variables. Standard descriptive statistics were used to describe H‐scores. H‐scores were dichotomized at their medians for each tumor compartment and categorized as low IGF2 expression if the H‐score was below the compartment median or high IGF2 expression if the H‐score was equal to or above the compartment median. Progression‐free (PFS) and overall survival (OS) were estimated using the Kaplan–Meier method and low‐ versus high IGF2 expression was compared using the log rank test. Univariable and multivariable Cox proportional hazard regression models were fit to examine the relationship of demographic variables and IGF2 expression to PFS and OS. Variables with *P *<* *0.10 in the univariable analysis were included in the multivariable model. All *P*‐values are two‐sided, and a *P*‐value of <0.05 was considered statistically significant. All analyses were performed using *Stata v13.0* (StataCorp, College Station, TX).

## Results

Clinicopathologic characteristics of the 103 patients who underwent primary surgical resection for uterine carcinosarcoma are shown in Table 1. Reflecting the known epidemiological risk factors for carcinosarcoma, patients were generally older, overweight or obese, and predominantly black.

**Table 1:** Demographic, pathologic, and treatment characteristics (*n* = 103)

Age (years)^a^	66.6 (11.2)	Adjuvant therapy	
BMI (kg/m^2^)^a^	34.6 (10.4)	No	47 (45.6)
BMI Categories		Yes	56 (54.4)
<18.5	1 (1.0)	Adjuvant chemotherapy	
18.5–24.9	12 (11.7)	No	66 (64.1)
25.0–29.9	19 (18.4)	Yes	37 (35.9)
30.0–34.9	17 (16.5)	Adjuvant radiotherapy	
35.0–39.9	13 (12.6)	No	83 (80.6)
≥40.0	23 (22.3)	Yes	20 (19.4)
Missing	18 (17.5)	Type of adjuvant RT	
Race/ethnicity		EBRT	9 (45.0)
White	36 (35.0)	EBRT + brachytherapy	7 (35.0)
Black	66 (64.1)	Brachytherapy only	3 (15.0)
Other	1 (0.9)	Other	1 (5.0)
FIGO Stage		IGF2 H‐scores^b^	
I	42 (40.8)	Epithelial nuclear	65 [0–285]
II	12 (11.6)	Epithelial cytoplasmic	180 [60–300]
III	30 (29.1)	Stromal nuclear	60 [0–200]
IV	19 (18.5)	Stromal cytoplasmic	160 [20–300]
Mesenchymal elements			
Homologous	47 (45.6)		
Heterologous	45 (43.7)		
Missing	11 (10.7)		
Residual tumor			
No	82 (79.6)		
Yes	20 (19.5)		
N/A	1 (0.9)		

a Reported as mean (standard deviation).

b Reported as median [Range].

Approximately half of patients had early stage (FIGO Stage I or II) and half had advanced stage (FIGO Stage III or IV) UCS. Most patients received adjuvant therapy following their surgery.

The median H‐score for IGF2 staining was higher in the cytoplasmic compartment compared to the nuclear compartment in both the malignant epithelial and malignant stromal tissue (Table 1). In less than 20% of cases, either malignant epithelium or stroma was absent from the stained section; therefore, missing H‐scores by compartment were as follows: epithelial nuclear and cytoplasmic *n* = 9, and stromal nuclear and cytoplasmic *n* = 10. Representative images of IGF2 immunohistochemical staining are shown in Figure 1. Histograms depicting the H‐score distribution in each tissue component are shown in Figure S1. As the H‐scores were not normally distributed, the median values rather than the mean values were selected as the optimal cutoff for defining high‐ versus low IGF2 expression. A moderate positive correlation was observed between IGF2 EN and IGF2 SN (Spearman's *ρ* = 0.65, *P* < 0.05), and between IGF2 EC and IGF2 SC (Spearman's *ρ* = 0.39, *P* < 0.05).

### Progression‐free survival

**Figure 2:**
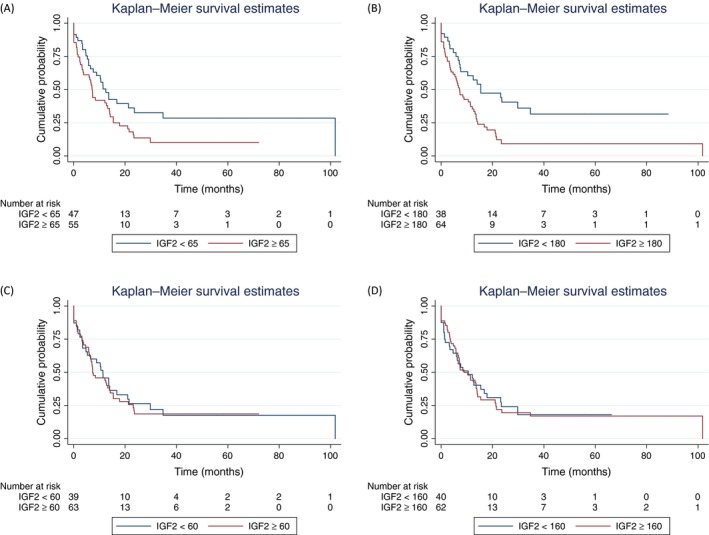
IGF2 expression and progression‐free survival (PFS). Kaplan–Meier curves depict the PFS in patients with high IGF2 expression (red line) versus low IGF2 expression (blue line), measured in the following malignant tissue compartments: epithelial nuclear (A), epithelial cytoplasmic (B), stromal nuclear (C), and stromal cytoplasmic (D). Patients with high‐CS epithelial IGF2 expression had reduced PFS compared with patients with low epithelial IGF2 expression (Log rank test *P *=* *0.036 and *P *=* *0.002 for epithelial nuclear and cytoplasmic compartments, respectively). Stromal IGF2 expression was not associated with PFS (Log rank test *P *=* *0.79 and *P *=* *0.91 for nuclear and cytoplasmic compartments, respectively).

Over the course of follow‐up (median 11.4 months), 75 patients had recurrence or progression of disease. The median PFS was 10.3 months [IQ Range: 3.2–23.2 months]. Progression‐free survival curves by tissue/cellular CS compartment are depicted in Figure 2. Patients with high‐CS epithelial IGF2 expression had reduced PFS compared with patients with low epithelial IGF2 expression (Log rank test *P *=* *0.036 and *P *=* *0.002 for epithelial nuclear and cytoplasmic compartments, respectively). Malignant stromal IGF2 expression was not associated with PFS (Log rank test *P *=* *0.79 and *P *=* *0.91 for stromal nuclear and cytoplasmic compartments, respectively).

The univariable and multivariable Cox proportional hazard's regression models for PFS are shown in Table 2. In univariable regression, black race, higher stage, high epithelial nuclear and cytoplasmic IGF2 expression were associated with an increased hazard of disease progression. Any adjuvant therapy was associated with a decreased hazard of progression of disease. In the multivariable model, after controlling for race, stage and adjuvant therapy (specifically chemotherapy), high IGF2 expression in both the epithelial nuclear and cytoplasmic compartments was associated with roughly twice the hazard of progression compared with low IGF2 expression (Epithelial nuclear HR = 1.80, 95% CI: 1.08–2.99, *P *=* *0.02 and epithelial cytoplasmic HR = 2.07, 95% CI: 1.19–3.60, *P *=* *0.01). After adjustment for IGF2 and stage, neither race, nor adjuvant therapy or chemotherapy, was independently associated with PFS.

**Table 2:** Univariable and multivariable Cox Proportional hazards models

Variables	Progression‐free survival		Overall survival	
	Univariable Hazard Ratio (95% CI, *P*‐value)	Multivariable Hazard Ratio (95% CI, *P*‐value)	Univariable Hazard Ratio (95% CI, *P*‐value)	Multivariable Hazard Ratio (95% CI, *P*‐value)
Age (years)	1.01 (0.99–1.03, 0.60)		1.02 (0.99–1.04, 0.10)	
BMI (kg/m^2^)	0.99 (0.97–1.02, 0.56)		0.98 (0.95–1.01, 0.22)	
Race				
White	1.00 (reference)	1.00 (reference)	1.00 (reference)	1.00 (reference)
Black	1.79 (1.06–3.02, 0.03)	1.68 (0.97–2.93, 0.07)	1.78 (1.04–3.06, 0.04)	1.68 (0.94–3.00, 0.08)
FIGO Stage				
I/II	1.00 (reference)	1.00 (reference)	1.00 (reference)	1.00 (reference)
III/IV	2.27 (1.41–3.65, <0.01)	2.11 (1.29–3.47, <0.01)	2.25 (1.39–3.67, <0.01)	2.28 (1.35–3.87, <0.01)
Sarcoma type				
Homologous	1.00 (reference)		1.00 (reference)	
Heterologous	1.10 (0.68–1.79, 0.70)		0.95 (0.57–1.58, 0.84)	
Adjuvant therapy ‐ any				
No	1.00 (reference)	1.00 (reference)	1.00 (reference)	1.00 (reference)
Yes	0.59 (0.37–0.94, 0.03)	0.55 (0.29–1.06, 0.07)	0.50 (0.31–0.80, <0.01)	0.40 (0.20–0.81, 0.01)
Adjuvant chemotherapy				
No	1.00 (reference)	1.00 (reference)	1.00 (reference)	1.00 (reference)
Yes	0.64 (0.39–1.05, 0.08)	1.15 (0.58–2.28, 0.70)	0.59 (0.35–0.98, 0.04)	1.15 (0.55–2.41, 0.71)
Adjuvant radiation				
No	1.00 (reference)		1.00 (reference)	
Yes	0.83 (0.47–1.46, 0.51)		0.67 (0.37–1.24, 0.20)	
Epithelial nuclear IGF2				
H‐score < median (65)	1.00 (reference)	1.00 (reference)	1.00 (reference)	1.00 (reference)
H‐score ≥ median (65)	1.64 (1.02–2.64, 0.04)	1.80 (1.08–2.99, 0.02)	1.72 (1.05–2.79, 0.03)	1.86 (1.10–3.15, 0.02)
Epithelial cytoplasmic IGF2				
H‐score < median (180)	1.00 (reference)	1.00 (reference)	1.00 (reference)	1.00 (reference)
H‐score ≥ median (180)	2.17 (1.30–3.62, <0.01)	2.07 (1.19–3.60, 0.01)	2.11 (1.25–3.57, <0.01)	1.98 (1.11–3.54, 0.02)
Stromal nuclear IGF2				
H‐score < median (60)	1.00 (reference)		1.00 (reference)	
H‐score ≥ median (60)	1.07 (0.66–1.71, 0.79)		1.04 (0.64–1.70, 0.86)	
Stromal cytoplasmic IGF2				
H‐score < median (160)	1.00 (reference)		1.00 (reference)	
H‐score ≥ median (160)	1.03 (0.64–1.65, 0.91)		1.07 (0.66–1.75, 0.76)	

### Overall survival

**Figure 3:**
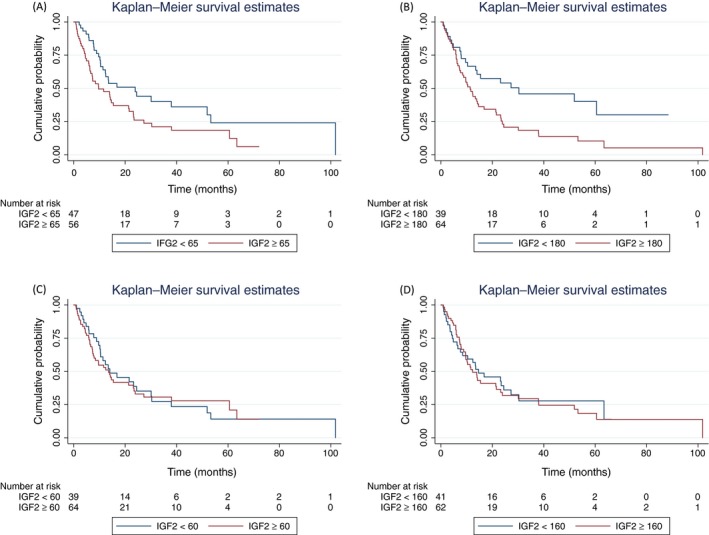
IGF2 expression and overall survival (OS). Kaplan–Meier curves depict the OS for patients with high IGF2 expression (red line) versus low IGF2 expression (blue line), as measured in the following malignant tissue compartments: epithelial nuclear (A), epithelial cytoplasmic (B), stromal nuclear (C), and stromal cytoplasmic (D). OS was significantly lower in women with high‐CS epithelial IGF2 expression (Log rank test *P *=* *0.03 and *P *=* *0.005 for epithelial nuclear and cytoplasmic compartments, respectively). Stromal IGF2 expression was not associated with OS (Log rank test *P *=* *0.86 and *P *=* *0.76 for stromal nuclear and cytoplasmic compartments, respectively).

Seventy patients died of disease by the end of follow‐up. The median OS was 13.9 months [IQ range: 6.23–51.9 months]. Overall survival curves by tissue/cellular CS compartment are depicted in Figure 3. Similar to PFS, OS was significantly lower in women with high‐CS epithelial IGF2 expression (Log rank test *P *=* *0.03 and *P *=* *0.005 for epithelial nuclear and cytoplasmic compartments, respectively). Malignant stromal IGF2 expression was not associated with OS (Log rank test *P *=* *0.86 and *P *=* *0.76 for stromal nuclear and cytoplasmic compartments, respectively).

The univariable and multivariable Cox proportional hazard's regression models for OS are shown in Table 2. In univariable regression models, black race, higher stage, and high epithelial nuclear and cytoplasmic IGF2 expression were associated with an increased risk of death. Any adjuvant therapy was associated with a decreased risk of death. In the multivariable model, after controlling for race, stage and adjuvant therapy, high IGF2 expression in both the epithelial nuclear and cytoplasmic compartments was associated with roughly twice the risk of death compared with low IGF2 expression (Epithelial nuclear HR = 1.86, 95% CI: 1.10–3.15, *P *=* *0.02 and epithelial cytoplasmic HR = 1.98, 95% CI: 1.11–3.54, *P *=* *0.02). Neither race nor adjuvant chemotherapy was independently associated with OS, after adjustment for IGF2 and stage.

### Stage and race

After stratifying patients by FIGO stage, high epithelial cytoplasmic expression of IGF2 in early‐stage UCS (stage I/II) was associated with an increased risk of disease progression (HR = 2.18, 95% CI: 1.05–4.54, *P *=* *0.04) and death (HR = 3.41, 95% CI: 1.47–7.90, *P *<* *0.01) after adjusting for race and adjuvant therapy (Table 3). Additionally, in women with early‐stage disease, black race was an independent poor prognostic factor for progression of disease (HR.2.83, 95% CI: 1.21–6.64, *P *=* *0.02) and death (HR = 2.80, 95% CI: 1.06–7.39; *P *=* *0.04). In patients with Stage III/IV disease, neither race nor IGF2 expression significantly impacted PFS or OS.

**Table 3:** Multivariable Cox proportional hazards models stratified by FIGO stage

Variables	Stage I/II		Stage III/IV	
	PFS hazard ratio (95% CI, *P*‐value)	OS hazard ratio (95% CI, *P*‐value)	PFS hazard ratio (95% CI, *P*‐value)	OS hazard ratio (95% CI, *P*‐value)
Race				
White	1.00 (reference)	1.00 (reference)	1.00 (reference)	1.00 (reference)
Black	2.83 (1.21–6.64, 0.02)	2.80 (1.06–7.39, 0.04)	0.96 (0.42–2.18, 0.92)	1.20 (0.53–2.74, 0.66)
Adjuvant therapy ‐ any				
No	1.00 (reference)	1.00 (reference)	1.00 (reference)	1.00 (reference)
Yes	0.52 (0.19–1.43, 0.21)	0.37 (0.12–1.19, 0.10)	0.45 (0.17–1.20, 0.11)	0.28 (0.10–0.76, 0.01)
Adjuvant chemotherapy				
No	1.00 (reference)	1.00 (reference)	1.00 (reference)	1.00 (reference)
Yes	0.77 (0.25–2.36, 0.65)	0.64 (0.18–2.32, 0.50)	1.86 (0.73–4.74, 0.19)	1.77 (0.66–4.74, 0.26)
Epithelial Nuclear IGF2				
H‐score < median (65)	1.00 (reference)	1.00 (reference)	1.00 (reference)	1.00 (reference)
H‐score ≥ median (65)	1.74 (0.78–3.86, 0.18)	1.50 (0.64–3.53, 0.36)	1.45 (0.69–3.03, 0.32)	1.81 (0.84–3.90, 0.13)
Epithelial Cytoplasmic IGF2				
H‐score < median (180)	1.00 (reference)	1.00 (reference)	1.00 (reference)	1.00 (reference)
H‐score ≥ median (180)	2.18 (1.05–4.54, 0.04)	3.41 (1.47–7.90, <0.01)	2.11 (0.88–5.07, 0.10)	1.10 (0.48–2.52, 0.81)

In FIGO stage I/II disease, IGF2 expression was higher in the EC compartment of those with black race (Median H‐score = 180, IQR: 160–190) compared to white race (Median H‐score = 160, IQR: 80–180), and this was significantly different (Wilcoxon Rank Sum test, *P *=* *0.02). Median H‐scores did not differ between racial groups for the other tissue/cellular compartments (EN, or either stromal compartment).

After stratification of patients by race, high IGF2 in the EC or the EN compartments was independently associated with worse survival in black women with UCS. A multivariable analysis was performed, including variables with *P *<* *0.1 on univariable analysis (Table S1). Adjusting for stage and adjuvant therapy, the race‐stratified HR for black women was 2.43 (95% CI: 1.18–5.01, *P *=* *0.02) for high IGF2 in the EC, and the HR was 2.34 for high IGF2 in the EN (95% CI: 1.25–4.39, *P *=* *0.008), as shown in Table 4.

**Table 4:** Cox stratified by race: Multivariable (keeping *P* < 0.10)

Variable	White			Black		
	Hazard Ratio	95% CI	*P*‐value	Hazard Ratio	95% CI	*P*‐value
Stage						
I/II	1.00 (ref.)		<0.001	1.00 (ref.)		0.04
III/IV	8.58	2.59–28.42		1.95	1.04–3.66	
Adjuvant therapy ‐ Any						
No	1.00 (ref.)		0.001	1.00 (ref.)		0.02
Yes	0.15	0.05–0.47		0.48	0.27–0.87	
Epithelial Nuclear IGF2						
H‐Score <median (65)				1.00 (ref.)		0.008
H‐Score >median (65)				2.34	1.25–4.39	
Epithelial Cytoplasmic IGF2						
H‐Score <median (180)				1.00 (ref.)		0.02
H‐Score >median (180)				2.43	1.18–5.01	

## Discussion

In this study, we found that elevated IGF2 protein expression is an independent poor prognostic factor in UCS. For patients whose tumor epithelial compartment demonstrated IGF2 expression equal to or above the median for the cohort, the risk of disease progression or death was approximately doubled. We also found that black women had higher tumor epithelial IGF2 expression compared to white women, and that high IGF2 was independently associated with worse survival in black women. Conversely, the impact of race on PFS and OS was attenuated, and no longer significant, after adjustment for IGF2 and stage. These novel findings suggest that tumor epithelial IGF2 expression is not only a prognostic biomarker in UCS, but also that IGF2 expression differences may contribute to the racial disparity in disease outcome in patients with UCS.

Unlike tumor epithelial IGF2 expression, the malignant stromal IGF2 expression was not prognostic of disease progression or survival. Recent studies of UCS show a high level of clonality, favoring a metaplastic origin of the sarcomatous elements derived from a common epithelial precursor as the carcinomatous elements 2, 5. Interestingly, metastatic implants of UCS are composed of purely carcinomatous elements in the majority of cases (69%), while 25% of cases have biphasic metastases and only 4% of cases have purely sarcomatous metastases 20. These observations support the primacy of the carcinomatous component as the driver of the UCS phenotype, consistent with the findings of the present study.

The racial disparity in survival observed in this study is consistent with a prior analysis of a clinical UCS cohort, a subset of which comprises the IGF2 study cohort. As previously reported in a retrospective review of 158 patients treated for UCS at a single institution, outcomes were worse in black women compared to white women with early‐stage disease after adjustment for adjuvant therapy 7.

Uterine corpus cancer is among the cancers with the largest racial disparity in survival. The five‐year survival rate is 86% in white women diagnosed with uterine cancer compared with 66% in black women. In multiple other cancers, including prostate, breast, and colorectal cancer, higher death rates are observed in blacks compared with whites in the United States 21. Socioeconomic status and discrimination significantly contribute to racial disparity in cancer survival, including endometrial cancer outcomes 22. Aggressive histologic types of endometrial cancer such as UCS occur more frequently in blacks, implicating tumor biology as a contributing factor to differences in mortality rates 23.

As in endometrial cancer, the aggressive types of breast cancer are more frequent in black women. A recent analysis of The Cancer Genome Atlas (TCGA) breast cancer cohort estimated that inherited germline variants account for 44% of racial differences in breast cancer subtype distribution 24. Comparison of blacks and whites defined by genomically determined genetic ancestry identified not only differentially expressed genes and proteins, but also significantly altered DNA methylation sites in breast cancer tissue. However, an earlier study of endometrial cancers from black and white women did not reveal significant differences in gene transcription, but was limited by the small number of patients 25. Thus, nonbiological and biological factors likely contribute to racial disparity in uterine cancer survival.

While race is a social construct, self‐identified race moderately correlates with genetic ancestry. It has been reported that metabolic variables such as insulin resistance preferentially cluster with genetic ancestry 26. The tumor necrosis factor (TNF)‐*α* axis, a cytokine initiator of inflammation, shows differences in genotype and allele frequencies (in TNF‐*α* and its receptor genes TNFR1 and TNFR2) between blacks and whites 27. Interestingly, TNF‐*α* has been identified as a regulator of IGF2 production by adipocytes 28.

Nonbiological factors may result in identifiable changes in downstream biological mediators, wherein stress‐inducing conditions, nutrition, and environmental toxins alter epigenetic markers and contribute to health disparities 29. For example, increasing poverty and nonwhite race is associated with elevated C‐reactive protein (CRP) levels 30. Of note, IGF2 is a regulator of CRP, and IGF2 was among the first genes identified to be altered in expression as a result of fetal exposure to poor nutrition 31. In addition, oxidative stress induces IGF2 overexpression via loss of imprinting triggered by NF‐*κ*B activation 32.

*IGF2* is one of a relatively small number of imprinted genes in mammals, by which epigenetic silencing results in monoallelic expression specific to parental origin 10. Most imprinted genes have key functions during embryonic development as is the case for *IGF2*. Upregulation of *IGF2* occurs in both childhood and adult malignancies, and its overexpression is linked to an aggressive phenotype of ovarian and prostate cancer 17, 33. Several important *IGF2* regulatory mechanisms have been identified, many of which overlap with functions during fetal development, including altered transcription factor expression, epigenetic changes such as altered DNA methylation, as well as changes in post‐transcriptional and post‐translational modulators of IGF2 expression 10.

Epigenetic events, particularly DNA methylation changes, are triggered by environmental factors and are key drivers of cancer development and progression 34. Differences in methylation status have been previously linked to racial disparity in breast, prostate, colorectal, and endometrial cancers 35. The increased tumor expression of IGF2 observed in black women with UCS may reflect differences in DNA methylation at its regulatory sites. We speculate that IGF2 expression is a potential biomarker of prior environmental exposures and stressors as well as heritable variations in metabolic and inflammatory pathways which interact to regulate IGF2 and cancer mortality in UCS.

Our group has previously shown that increased tumor expression of IGF2 in epithelial ovarian cancer cells is associated with resistance to paclitaxel and other microtubule stabilizing drugs, and that IGF‐targeting therapy restored drug sensitivity in cell line and xenograft models of ovarian cancer 17, 36. Other groups have shown that IGF2 overexpression is associated with resistance to chemotherapy agents such as cisplatin, adriamycin, and fluorouracil 37, 38, 39. As paclitaxel and platinum drugs comprise the most commonly used adjuvant treatment for UCS, modulation of chemosensitivity by IGF2 is highly clinically relevant. Our laboratory is presently evaluating the ability of IGF2‐targeting therapy to enhance efficacy of chemotherapy using primary UCS cell lines and patient‐derived UCS xenografts.

In summary, we have identified IGF2 protein expression as an independent poor prognostic biomarker in UCS, a lethal uterine cancer that disproportionately impacts black women. These novel findings, in conjunction with prior studies of IGF2 in cancer, further substantiate that IGF2 is a biological mediator of an aggressive cancer phenotype and a potential therapeutic target. Moreover, we have uncovered a novel potential association of IGF2 with racial disparity in UCS, which merits further investigation.

## Conflicts of Interest

The authors declare that there are no conflict of interests.

## Supporting information


**Figure S1**. Histograms for H‐score distribution for each malignant tissue compartment.

**Table S1**. Cox stratified by Race: Univariable.
