# Supplementary material for: Insulin‐like growth factor 2: a poor prognostic biomarker linked to racial disparity in women with uterine carcinosarcoma
Source: Cancer Med. 2018 Feb 18;7(3):616–25. doi: 10.1002/cam4.1335 (PMC5852335; doi:10.1002/cam4.1335)

Supplemental Figure S1.
Histograms for H-score distribution for each malignant tissue compartment.

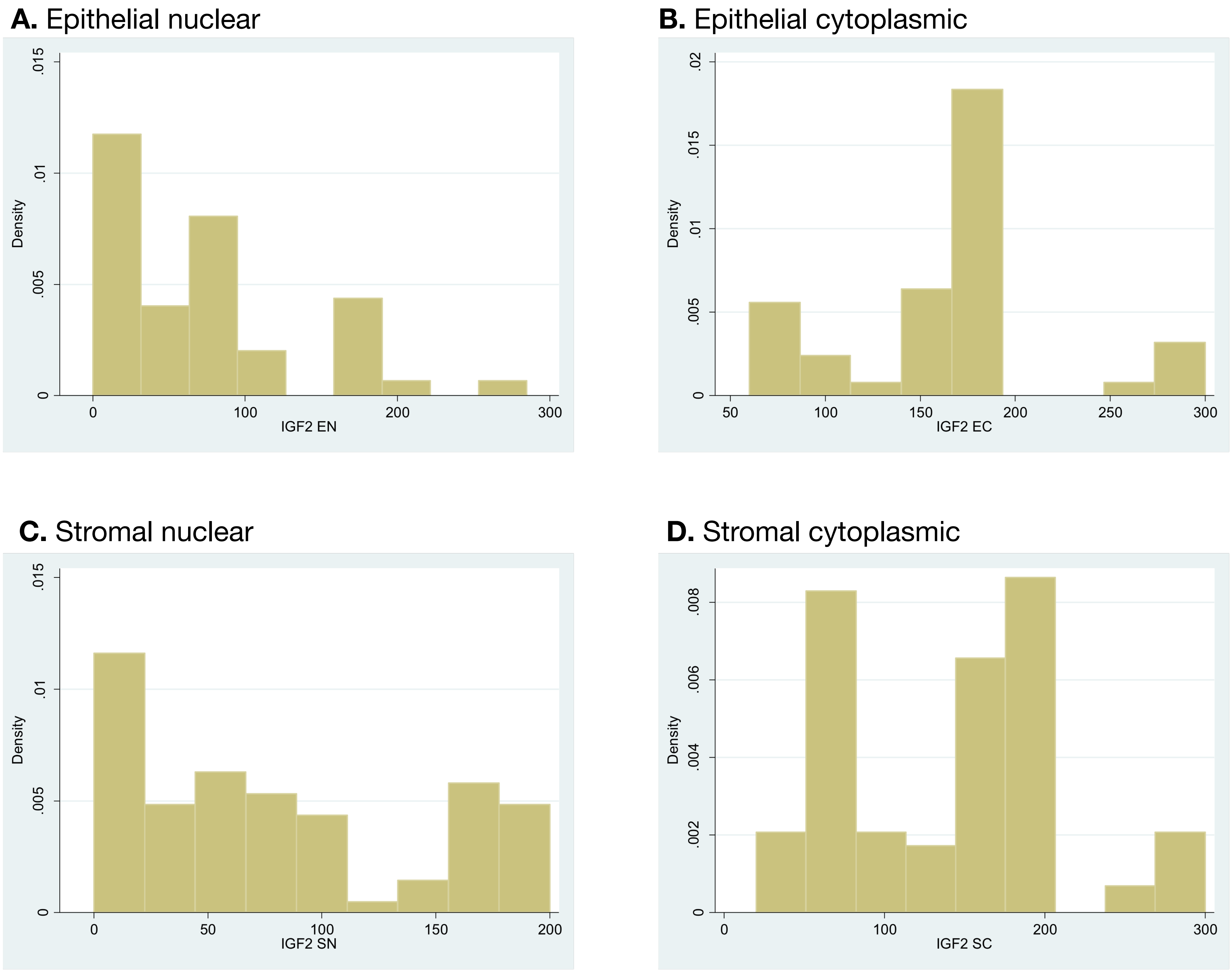

Supplement: Supplementary file 1 — Figure S1. Histograms for H‐score distribution for each malignant tissue compartment. [file CAM4-7-616-s001.pdf]
