# Supplementary material for: Insulin‐like growth factor 2: a poor prognostic biomarker linked to racial disparity in women with uterine carcinosarcoma
Source: Cancer Med. 2018 Feb 18;7(3):616–25. doi: 10.1002/cam4.1335 (PMC5852335; doi:10.1002/cam4.1335)
Supplement: Supplementary file 2 — Table S1. Cox stratified by Race: Univariable. [file CAM4-7-616-s002.docx]

Table S1. Cox stratified by Race: Univariable

|  | White | | | Black | | |
| --- | --- | --- | --- | --- | --- | --- |
| Variable | Hazard Ratio | 95% CI | p-value | Hazard Ratio | 95% CI | p-value |
|  |  |  |  |  |  |  |
| Age (years) | 1.02 | 0.99-1.06 | 0.22 | 1.01 | 0.99-1.04 | 0.33 |
|  |  |  |  |  |  |  |
| BMI (kg/m^2^) | 0.97 | 0.92-1.03 | 0.33 | 0.99 | 0.96-1.03 | 0.61 |
|  |  |  |  |  |  |  |
| Stage |  |  | 0.008 |  |  | 0.04 |
| I/II | 1.00 (ref.) |  |  | 1.00 (ref.) |  |  |
| III/IV | 4.02 | 1.43-11.33 |  | 1.85 | 1.05-3.30 |  |
|  |  |  |  |  |  |  |
| Adjuvant therapy - Any |  |  | 0.04 |  |  | 0.01 |
| No | 1.00 (ref.) |  |  | 1.00 (ref.) |  |  |
| Yes | 0.38 | 0.15-0.96 |  | 0.49 | 0.28-0.86 |  |
|  |  |  |  |  |  |  |
| Adjuvant Chemotherapy |  |  | 0.12 |  |  | 0.12 |
| No | 1.00 (ref.) |  |  | 1.00 (ref.) |  |  |
| Yes | 0.44 | 0.16-1.24 |  | 0.62 | 0.34-1.13 |  |
|  |  |  |  |  |  |  |
| Adjuvant Radiation |  |  | 0.97 |  |  | 0.08 |
| No | 1.00 (ref.) |  |  | 1.00 (ref.) |  |  |
| Yes | 0.98 | 0.35-2.76 |  | 0.50 | 0.23-1.07 |  |
|  |  |  |  |  |  |  |
| Adjuvant Chemo + RT |  |  | 0.42 |  |  |  |
| No | 1.00 (ref.) |  |  | 1.00 (ref.) |  |  |
| Yes | 2.31 | 0.30-17.98 |  | -- | -- |  |
|  |  |  |  |  |  |  |
| Epithelial Nuclear IGF2 |  |  | 0.16 |  |  | 0.03 |
| H-Score <median (65) | 1.00 (ref.) |  |  | 1.00 (ref.) |  |  |
| H-Score >median (65) | 2.11 | 0.75-5.94 |  | 1.86 | 1.06-3.27 |  |
|  |  |  |  |  |  |  |
| Epithelial Cytoplasmic IGF2 |  |  | 0.11 |  |  | 0.05 |
| H-Score <median (180) | 1.00 (ref.) |  |  | 1.00 (ref.) |  |  |
| H-Score >median (180) | 2.17 | 0.84-5.65 |  | 1.90 | 1.00-3.59 |  |
|  |  |  |  |  |  |  |
| Stromal Nuclear IGF2 |  |  | 0.75 |  |  | 0.67 |
| H-Score <median (60) | 1.00 (ref.) |  |  | 1.00 (ref.) |  |  |
| H-Score >median (60) | 1.17 | 0.44-3.14 |  | 1.13 | 0.65-1.98 |  |
|  |  |  |  |  |  |  |
| Stromal Cytoplasmic IGF2 |  |  | 0.81 |  |  | 0.53 |
| H-Score <median (160) | 1.00 (ref.) |  |  | 1.00 (ref.) |  |  |
| H-Score >median (160) | 1.12 | 0.43-2.90 |  | 1.20 | 0.68-2.11 |  |
